# Distribution pattern of colorectal diseases based on 2300 total colonoscopies 

**Published:** 2017

**Authors:** Yousef Bafandeh, Fereshteh Yazdanpanah

**Affiliations:** 1 *Division of Gastroenterology, Imam Reza Hospital, Tabriz University of Medical Sciences, Tabriz, Iran*; 2 *Faculty of Medicine, Tabriz University of Medical Sciences, Tabriz, Iran*

**Keywords:** Colorectal disease, Colonoscopy, Prevalence, East Azerbaijan, Iran

## Abstract

**Aim:**

We conducted a cross-sectional study to estimate the prevalence of CRD in our area in East Azerbaijan, Iran.

**Background:**

Colorectal diseases (CRD) include a broad spectrum that varies from benign lesions to malignant and cancerous masses. CRD can be investigated by colonoscopy. Some of these diseases are highly preventable with timely screening and appropriate planning by the healthcare system.

**Methods:**

This is a hospital-based, cross-sectional study by the Gastroenterology Department on patients who underwent colonoscopy between June 2013 and March 2015 in outpatient clinics at private Shahriyar medical center and Imam Reza University Hospital in Tabriz, East Azerbaijan province, Iran. Chi-square analysis and bivariate Pearson correlation coefficient were applied in this study using SPSS 20.

**Results:**

During the study period, we recruited 2300 patients (1230 men, 1070 women) with the mean and median (Standard Deviation=SD) age at presentation of 47.10 (0.338) and 47 (16.195) years. The duration of patients’ symptoms ranged from 1 to 480 months, with mean and median (SD) values of 26.99 (0.902) and 12 (40.76) months, respectively. Despite at least 20 types of pathologies seen on colonoscopy, normal cases constituted the majority (32.7%).The most serious diseases in the study were IBD (10.9%), polyps (14.4%) and cancers (4.9%). Colonoscopic findings had a significant correlation with patients’ age and gender; also, we detected a significant correlation between patients’ chief complaint and colonoscopic findings as well as colonoscopic pathology samples.

**Conclusion:**

Despite the ongoing westernization of lifestyle in our country, the distribution of colorectal diseases in Iran is different from Western population. It is important to recognize the prevalence of these diseases in our area to determine exposure factors for management and planning correctly in health system policies.

## Introduction

 Colorectal diseases (CRD) include a broad spectrum that varies from benign lesions to malignant and cancerous masses. CRD incidence and mortality rates vary markedly around the world with global differences in risk factors such as age, race, sedentary lifestyle, unhealthy diet, smoking, obesity, alcohol, positive family history and radiation therapy. CRD have a broad spectrum: ileitis, colitis, erosion, angiodysplasia, diverticula, infiltrative lesions, inflammatory bowel disease (IBD), pneumatosis coli, familial adenomatous polyposis (FAP), amebiasis, various types of polyps, cancer, fissure, fistula, hemorrhoids, rectal varices, etc, which can be investigated by colonoscopy. Colonoscopy may be carried out for diagnostic and/or therapeutic reasons. It is performed for screening or surveillance for colon cancer, evaluating signs and symptoms suggestive of possible colonic or distal small bowel disease, assessing a response to treatment in patients with known colonic disease (e.g., IBD), and evaluating abnormalities found on imaging studies (1). The procedure is a safe and effective means of evaluating the large bowel. The risk of serious complications, including perforation and bleeding, is low with a mortality rate of 0.007% (2). The technology for colonoscopy has evolved to provide a very clear image of the mucosa through a video camera attached to the end of the scope. The camera connects to a computer, which can store and print color images selected during the procedure. Screening for and follow-up of colorectal cancer (CRC) are among the indications for colonoscopy (1). Although CRC is highly preventable, it is the second most common cancer and cause of cancer death in the United States, where the lifetime incidence of CRC in patients at average risk is about 5% (3). It is the third most common cancer worldwide and the fourth most common cause of death [3]. Proper screening can help to reduce mortality rate at all ages, and colonoscopy plays an important role in this effort. Recommendations vary among the leading organizations in this field, namely the American Cancer Society (ACS), the World Health Organization (WHO), the US Preventive Services Task Force (USPSTF), and the American College of Physicians (ACP). It is generally recommended, however, that average-risk adults should begin CRC screening at age 50 years, utilizing one of several options for screening, including colonoscopy, every 10 years (4, 5). Similar to CRC, other CRD have different global incidence rates due to various factors. Considering the large geographic difference in the global distribution of CRC and other CRD, we performed a cross-sectional study to estimate the prevalence of CRD in our area in East Azerbaijan, Iran, as a similar study has not been done before in this area.

## Methods

This is a hospital-based, cross-sectional study by the Gastroenterology Department on 2300 patients who underwent total colonoscopy for different lower gastrointestinal (LGI) symptoms, between June 2013 and March 2015,conducted through outpatient clinics at private open access Shahriyar medical center and Imam Reza University Hospital in Tabriz, East Azerbaijan province, Iran. The inclusion criteria were all patients with lower GI symptoms referred for colonoscopy to the centers mentioned above. Cecal intubation was documented by video-CD recording. Ileal intubation was performed in cases with suspicious disorders. Withdrawal time was 6-10 minutes. The exclusion criteria were any absolute contraindication to colonoscopy and failure to reach the cecum. Among polyp cases, we performed polypectomy of polyps larger than 1cm and biopsied those smaller than 5mm. In cases with polyps 5-10mm in size, we decided based on symptoms, personal or family history and histopathologic findings. The participants of this study could be proper representative of the region community, as people who refer to the above centers are quite diverse in characteristics such as age, gender, socioeconomic, etc. The data were collected through questionnaires inquiring about the demographic data, chief compliant at the onset of the disease, time interval between the onset of the disease and diagnosis, and colonoscopy outcomes. The questionnaires were completed by trained paramedical personnel for all patients. The statistical methods used in this study included Chi-square analysis in order to determine the relationship between two categorical variables and bivariate Pearson correlation coefficient for comparative analysis of continuous variables. Statistical significance was determined by P values less than 0.05. All statistical processes were performed using SPSS version 20.

## Results

**Figure 1:**
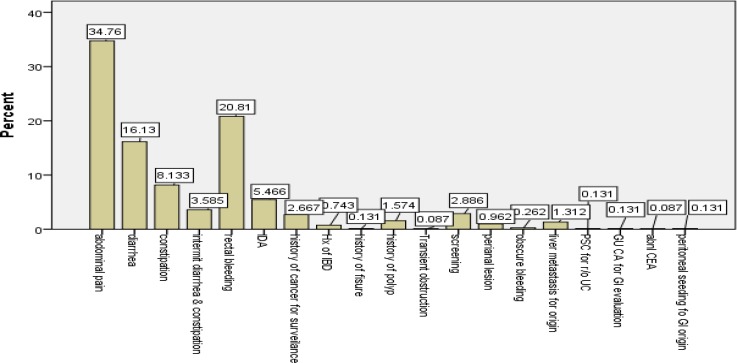
Chief complication of patients

**Figure 2:**
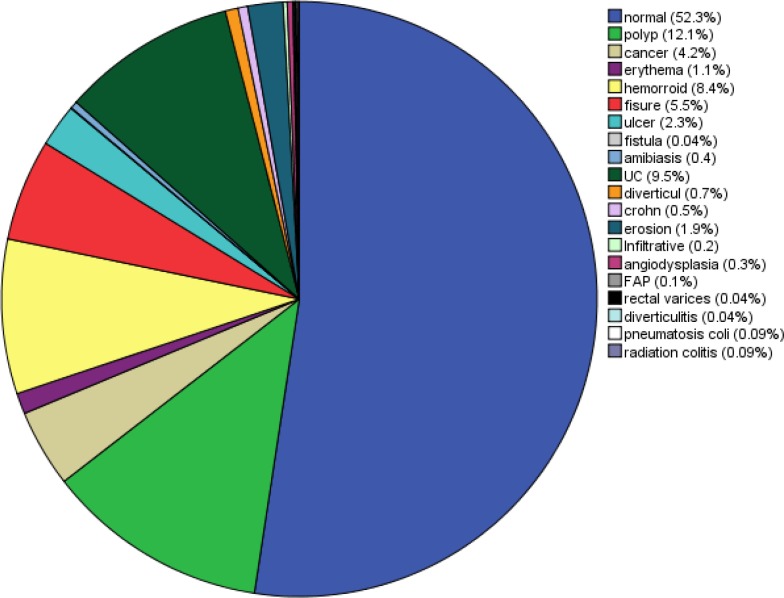
Secondry findings in total colonoscopy of investigated patients

**Figure 3:**
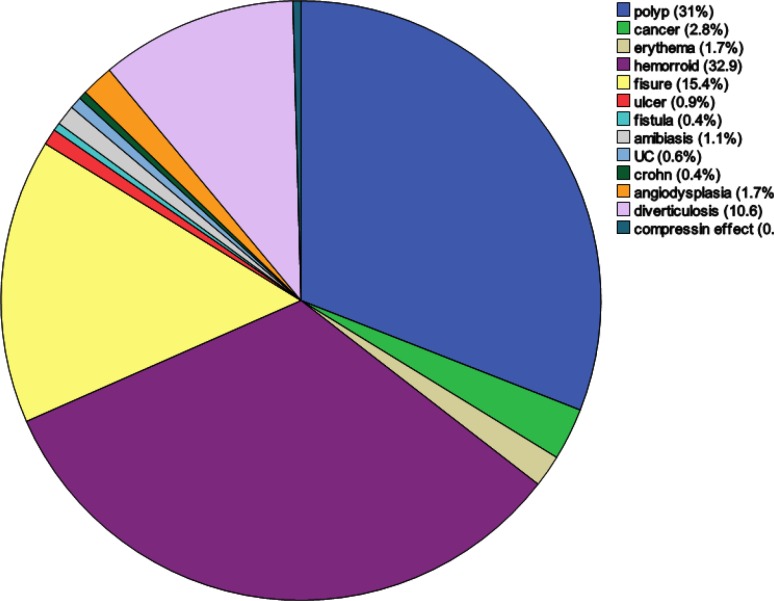
Main findings in 2300 total colonoscopy

During the study period, we recruited 2300 patients (1230 men, 1070 women) with confirmed indications for colonoscopy. The male to female ratio was 1.14:1. The patients’ age ranged from 12 to 120 years; and the mean and median (SD) age at presentation were 47.10 (0.338) and 47 (16.195) years, respectively. Among the study population, 84 (3.65%) were 20 years old or younger and the others (96.3%) were older than 20. The duration of patients’ symptoms ranged from 1 to 480 months, with mean and median (SD) values of 26.99 (0.902) and 12 (40.76) months, respectively. Indications for colonoscopy are represented in figures 1 and 2. Some patients had more than one complaint; therefore, the complaints were marked as chief (figure 1) or secondary (figure 2) complaints. Abdominal pain (41.2%), rectal bleeding (31.8%) and diarrhea (26.4%) were the most common indications. Colonoscopic findings are shown in figure 3.

**Figure 4:**
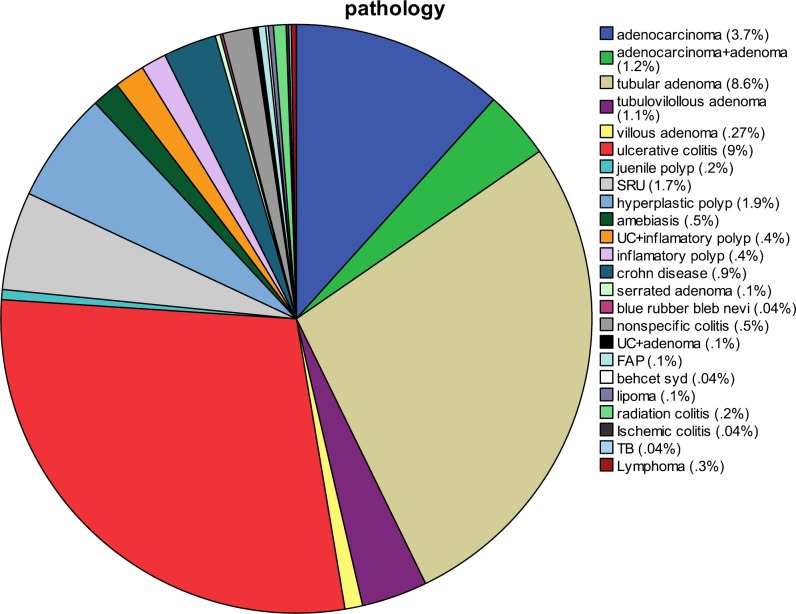
Histopathologic findings in patients underwent total colonoscopy

Findings explaining the chief complaint were marked as primary (3) and incidental findings as secondary (figure 2). Despite at least 20 types of pathologies seen on colonoscopy (figure 4), normal cases constituted the majority (32.7%); the gender distribution in this group was approximately equal, with 50.1% men and 49.9% women. The age range in the group with normal colonoscopy was very broad, from 13 to 101 years, with a mean value of 46.74 ± 15.64 years. The patients were referred for various reasons but most complained from chronic unexplained abdominal pain (48.3%) and diarrhea (16%). In addition, the number of UC and CD patients was 252 (10.9%). The ratio of ulcerative colitis: Crohn's disease was 18.25:1. There were 231 patients (10%) with ulcerative colitis, consisting of 123 males and 96 females (mean age 37.74 ± 14.07 years, median 35 years) and a male to female ratio of 1.28:1. There were 21 patients (0.5%) with Crohn's disease, consisting of 17 males and 4 females (mean age 34.75± 13.23 years, median age 28 years) with a male to female ratio of 1.2:1. Furthermore, rectal bleeding (33.0%) and abdominal pain (50.0%) were the most prevalent presentations in UC and CD patients, respectively. Cancer cases accounted for 4.9% of all patients (111 cases). In this category, there were 73 (66%) men and 38 (34%) women (mean age 58.90 ± 14.37 years, median age 60 years). In addition, rectal bleeding (33%) was the most common chief compliant in cancer patients, and rectum (38.1%) and sigmoid (19.6%) were the most affected locations. Polyps constituted 14.4% (332 cases) of lesions observed in colonoscopies. Tubular adenoma (75.5%) was the most common type, followed by hyperplastic polyps (15%), tubulovillous (2.7%), villous (2.7%) and others (12.8%), including FAP, Juvenile, mucosal tag and inflammatory types (figures 4). In this group with a mean age of 55.10 ± 14.15 years and a median of 56 years, the male to female ratio was 1.47:1. Abdominal pain (33.2%) and rectal bleeding (24.1%) were the most prevalent presentations; also, sigmoid (28.1%) and rectum (24.1%) were the most affected locations. In addition, we observed some insignificant lesions such as hemorrhoids (41.3%), perianal fissures (20.9%) and fistula (0.4%), amebiasis [histopathologically proven (1.7%)], diverticulosis (11.3%) and angiodysplasia (2%) (Figure 3).

The colonoscopic findings had a significant correlation with patients’ age and gender (p < 0.01, p = 0.014, respectively). Also, we detected a significant correlation between patients’ chief complaints and colonoscopic, as well as histopathologic findings (both p < 0.01). Another remarkable finding worthy of investigation is the significant correlation between chief complaint and lesion location on colonoscopy, as well as the association between types of lesion and lesion location (p value < 0.01). 

## Discussion

Colorectal disease (CRD) is a general term that refers to a wide range of colon and rectum disease. It is undoubtedly useful to recognize the prevalence of CRD in detail, as some of these diseases are lethal or irritating, they may be preventable and, with adherence to recommended screening guidelines, the risk of developing or dying from colorectal cancer may be reduced. The fact that some of the most important CRD, such as colorectal cancer (CRC) and inflammatory bowel disease (IBD), are known as problems of Western or industrial countries (6), as well as the fact that “Westernization” has increased the prevalence of obesity and decreased the level of physical activity in our country similar to many parts of the world, emphasizes the significance of these diseases and highlights the need for knowing their prevalence in our area to determine exposure factors as risk factors of these disease for management and planning correctly in health system policies. CRC is an important public health problem; every year, there are nearly one million new cases of colorectal cancer diagnosed worldwide with half a million deaths (7). In Iran, 5000 new cases are reported annually (8). Along with the significant increase of IBD all over the world, the results of recent studies have revealed increasing trends for both UC and CD in the past 3 decades in our country (9); therefore, the importance of screening and estimating the prevalence of this disease is clear. In our study, more than one third of patients who underwent colonoscopy and had a gastrointestinal chief compliant were normal. A normal colonoscopy is not considered significant, although this may be relevant to patient care as it may rule out a serious disease in the colon during surveillance.

There is poor correlation between colonic disorders and lower gastrointestinal (GI) symptoms. The American Society for Gastrointestinal Endoscopy appropriateness guideline (10) and the European panel guideline for appropriateness of gastrointestinal endoscopy (11) have appeared as potential solutions to tackle this problem and to increase detection rates of relevant lesions. Despite the potential role of appropriateness guidelines, they have not been widely adopted partly due to fear of missing significant lesions detected in inappropriate indications (12). Thus, the combination of clinical criteria (appropriateness or prioritizing criteria) with blood or fecal markers might be a better approach than isolated clinical criteria to increase the diagnostic yield of significant lesions.

Our study has shown an overall diagnostic yield of 67.3% (1548 positive findings out of 2300 cases). Diagnostic yield is defined as the ratio between significant findings detected on colonoscopy and the total number of procedures performed for that indication. This figure is different from the 79.0% diagnostic yield found by Ismaila and Misauno in Jos, Nigeria (13), studies in the West African sub-region carried out by Mbengue et al. (14), 48.0% obtained by Sahu et al. (15), amongst their Indian patients, the 27.2% found by Siddique et al. (16), and 21.0% reported by Al-shamali et al. (17) amongst the Saudis. The differences in the diagnostic yield may be due to varying sample sizes in the studies, the differences in the spectrum of colonic diseases seen in different regions of the world, and the different selection criteria and indications for colonoscopy.

Abdominal pain (41/2%), rectal bleeding (31/8%) and diarrhea (26/4%) were the most common indications. These symptoms, in addition to iron deficiency anemia (IDA), assessment of IBD, CRC, polyp screening and surveillance are the main indications for colonoscopy according to EPAGE II [11]. Patients with IDA and CRC accounted for 9.2% and 2.9%, respectively. In a Spanish study by Gimeno García et al. (18) on 1004 cases, the most common indication was colorectal cancer (CRC) screening (35.2 %). In an African study by Obonna G et al. (19) on 100 patient, indications were frank lower gastrointestinal bleeding 55 (55%) chronic diarrhea (11%), chronic constipation (10%), occult gastrointestinal bleeding (7.0%), lower abdominal and anal pain (4.0%), queried anorectal cancer (3.0%) and enterocutaneous fistula (1%).

The main reason for the differences in the spectrum of findings on colonoscopy may be due to racial differences, geographical variations, lifestyle and environmental, behavioral and dietary factors. In a study by Alatise OI et al. (20) on 320 cases, abnormal endoscopic findings included 66 (20.6%) patients who had hemorrhoids, 50 (15.6%) with colorectal cancer, 33 (10.3%) patients with benign polyps and 30 (9.4%) patients with diverticular disease. Other findings were colitis, inflammatory bowel disease, rectovaginal fistula, vascular ectasia and extra luminal compression.

 The most commonly seen lesion in our cross-sectional study was polyps (14.4%) of various types with the tubular type as predominant pathology, which is in line with previous studies (21). The most commonly seen location of polyps was sigmoid and rectum. The incidence rate of polyps varies in different age groups; however, it is obviously less prevalent in our population in comparison with Western countries, amounting to approximately 30% in asymptomatic adults. Like other studies, males have a modestly higher incidence than females and they lean towards the elderly with a mean age of 55.10 years. There is no difference in terms of most affected location in past studies (21). The second common lesion is Ulcerative colitis, one of the inflammatory bowel disease (IBD) types. In the current study, IBD accounted for 10.9% of all lesions seen on colonoscopy. The peak age at diagnosis showed a pattern similar to other Asian countries, such as India (22) and Japan (23). In this pattern, only one peak at the age range of 20-40 was documented and no second peak (>60 years old), usually observed in developed countries (24), was detected. On the other hand, two studies in South Korea (25) and Hong Kong (26) demonstrated have small second peaks in their elderly population. In other studies performed in Iran, the male to female ratio has been reported to be 0.8:1 (27) and 0.7:1 (28) for UC and 1.4:1 (29) and 1.2:1 (30) for CD patients. In addition, the female predominance for UC in those studies is similar to another study conducted in Iran (9) but in contrast to equal gender distribution for UC in the present study (1.2:1) and other Asian countries (31-33). The most prevalent presentation was rectal bleeding (33.0%) for UC and abdominal pain (50.0%) for CD patients. Despite the similarity between our results and other Asian (34) UC patients regarding their first chief complaint, a clear difference was observed between our population and other Iranian people who suffered from UC based on three studies conducted previously in Iran (9,27,28). It is interesting that most of our IBD cases (91.6%) have UC versus only 8.4% who have CD, indicating a considerable difference with studies on Western populations. Although the prevalence and incidence of IBD have not been properly studied in Iran, our country is among those with an increasing rate of IBD (9). Moreover, since previous studies did not use data from the national registry system and were more hospital-based and retrospective, no clear epidemiological data is available on the prevalence of IBD in Iran; nevertheless, in our study only 10.9% of symptomatic patients were affected with IBD which might be a considerable statistic, since IBD is classified as a rare disease.

Finally, we focused on cancerous lesions. In this study, men were affected with CRC more than women, nearly by two folds, which is in contrast to the symmetric sex distribution in the world population (35), but is almost similar to a previously performed study in our area in 2006 (36). The most common presentation for people in our region was rectal bleeding, similar to other studies. This study, like previous ones, shows that CRC is a disease of the elderly with a mean age of 58.90 years, although a previous study in our region shows a higher mean age (73 years) of diagnosis for CRC (35). The most commonly affected locations for cancer were rectum and sigmoid which is similar to global reports (37) but in contrast to the study by Manas Kotepui et al. This may be related to difference in population’s race. Considering the rapidly aging population in developing countries, the number of colorectal cancers diagnosed will increase in the years to come; therefore, it is essential to emphasize the increasing need for research in different aspects of prevention and planning for treatment of these patients in our developing country. 

This study investigates a general scheme of CRD prevalence in our local area, with a clearly lower than national average prevalence of CRC. We conclude that there is a significant association between colonoscopic findings and patients’ age and gender. Also, a significant correlation was observed between patients’ chief complaint and colonoscopy findings as well as colonoscopically sampled pathology. Another important point to investigate was the significant correlation between the chief complaint and lesion location on colonoscopy, as well as the association between types of lesion and lesion location. Although the large study population was one of the advantages of this study, it has multiple limitations and should be considered in future. First, our study was performed in only 2 open access hospitals from numerous healthcare centers in Tabriz; although one of them is the largest referral tertiary center in our region, it should be remembered that this study does not explicitly represent the entire population of the city or the country. Another limitation is that we did not check the individuals’ risk factors and exposures; therefore, we cannot evaluate the causes of disease prevalence which may limit planning by the healthcare system for exposure reduction and screening program for the population groups at risk. Due to the above mentioned limitations, we recommend a comprehensive study to investigate the exact CRD prevalence with associated risk factors.

It is concluded that in our country, the distribution pattern of colorectal diseases is different in comparison with Western populations, especially for IBD, polyps and CRC.

## Conflict of interest

The authors declare that they have no conflict of interest.
